# Transcriptional Regulation of Genes Involved in Zinc Uptake, Sequestration and Redistribution Following Foliar Zinc Application to *Medicago sativa*

**DOI:** 10.3390/plants10030476

**Published:** 2021-03-03

**Authors:** Alessio Cardini, Elisa Pellegrino, Philip J. White, Barbara Mazzolai, Marco C. Mascherpa, Laura Ercoli

**Affiliations:** 1 Institute of Life Sciences, Scuola Superiore Sant’Anna, 56127 Pisa, Italy; alessio.cardini@santannapisa.it (A.C.); laura.ercoli@santannapisa.it (L.E.); 2 Department of Ecological Science, The James Hutton Institute, Invergowrie, Dundee DD2 5DA, UK; philip.white@hutton.ac.uk; 3 Center for Micro-BioRobotics, Istituto Italiano di Tecnologia, Pontedera, 56025 Pisa, Italy; barbara.mazzolai@iit.it; 4 Istituto di Chimica dei Composti Organo Metallici, National Research Council (CNR), 56124 Pisa, Italy; marcocarlo.mascherpa@pi.iccom.cnr.it

**Keywords:** ZIP transporters, nicotianamine, metal tolerance protein (MTP), yellow stripe-like protein (YSL), zinc-induced facilitators (ZIF), heavy metal transporters (HMA)

## Abstract

Zinc (Zn) is an essential micronutrient for plants and animals, and Zn deficiency is a widespread problem for agricultural production. Although many studies have been performed on biofortification of staple crops with Zn, few studies have focused on forages. Here, the molecular mechanisms of Zn transport in alfalfa (*Medicago sativa* L.) were investigated following foliar Zn applications. Zinc uptake and redistribution between shoot and root were determined following application of six Zn doses to leaves. Twelve putative genes encoding proteins involved in Zn transport (*MsZIP1-7*, *MsZIF1*, *MsMTP1*, *MsYSL1*, *MsHMA4*, and *MsNAS1*) were identified and changes in their expression following Zn application were quantified using newly designed RT-qPCR assays. These assays are the first designed specifically for alfalfa and resulted in being more efficient than the ones already available for *Medicago truncatula* (i.e., *MtZIP1-7* and *MtMTP1*). Shoot and root Zn concentration was increased following foliar Zn applications ≥ 0.1 mg plant^−1^. Increased expression of *MsZIP2*, *MsHMA4*, and *MsNAS1* in shoots, and of *MsZIP2* and *MsHMA4* in roots was observed with the largest Zn dose (10 mg Zn plant^−1^). By contrast, *MsZIP3* was downregulated in shoots at Zn doses ≥ 0.1 mg plant^−1^. Three functional gene modules, involved in Zn uptake by cells, vacuolar Zn sequestration, and Zn redistribution within the plant, were identified. These results will inform genetic engineering strategies aimed at increasing the efficiency of crop Zn biofortification.

## 1. Introduction

A large proportion of the world’s population suffers from Zn-related diseases (i.e., malabsorption syndrome, liver disease, chronic renal disease, sickle cell disease, and other chronic diseases), since they rely on cereal-based diets with low Zn content due to poor soil Zn availability [1,2,3,4]. Diversification of the human diet and biofortification of edible crops are therefore needed to alleviate Zn deficiency in humans. Similarly to humans, animals can suffer from Zn deficiencies that could be alleviated by biofortified feed or Zn supplementation, thus improving livestock health and quality of food products, which affect human health indirectly [5,6,7,8].

Zinc plays a major role as a co-factor of over 300 enzymes in plants and is an essential micronutrient [9]. Zinc is involved in various physiological functions, such as CO_2_ fixation, protein synthesis, free radical capture, regulation of growth and development, and disease resistance [9,10]. Many structural motifs in transcriptional regulatory proteins are stabilized by Zn, such as Zn finger domains [11]. Zinc deficiency reduces crop production, as does Zn excess [12]. Excessive Zn^2+^ can compete with other cations in binding to enzymes and for transport across membranes, thereby impairing cellular activities [12]. Thus, the uptake of Zn^2+^ by cells and its transport within the plant must be strictly regulated. Plant cells have evolved several homeostatic mechanisms for avoiding Zn^2+^ toxicity when exposed to large Zn availability in their environment. These include the reduction of Zn influx to cells, the stimulation of Zn efflux from the cytosol, the sequestration of Zn in vacuoles, and the chelation of Zn by Zn-binding ligands. In general, the concentration of Zn in plant tissues must be kept between 15 and 300 µg Zn g^−1^ dry matter (DM) to maintain cell structure and function [12,13]. Although tolerance to large tissue Zn concentrations varies among species [12,14], Zn concentrations above 400–500 µg g^−1^ DM often cause toxicity symptoms including impaired root and shoot growth, chlorosis and necrosis of leaves, reduced photosynthesis, nutrient imbalance, and ultimately loss of yield [9,12,15,16].

The process of producing crops with greater mineral concentrations in edible tissues is called biofortification and provides a solution to the problem of mineral deficiencies in human and animal nutrition [17]. There are various approaches to Zn biofortification of edible crops, including agronomic strategies and conventional or transgenic breeding strategies. Agronomic biofortification aims to increase Zn concentrations in edible tissues through the application of Zn fertilizers to the soil or to leaves. It is relatively inexpensive and efficient [18]. Foliar application of Zn is generally more effective than the application of Zn fertilizers to soil, since Zn uptake by plant roots is often limited by the low solubility of Zn salts, its binding to organic substrates, and its immobilization in the microbial biomass [19]. Both agronomic and genetic biofortification strategies have been studied extensively in cereal staple crops, such as rice, wheat, and maize, but less in legumes, such as beans, peas, or lentils [17,20,21]. An international program, the HarvestPlus Zinc Fertilizer Project, is exploring the potential of Zn fertilizers to enhance the yields and Zn concentrations in edible portions of staple crops in developing countries of Africa, Asia, and South America (www.harvestzinc.org (accessed on 2 March 2021)) [22], but this program does not include forage crops. 

The natural direction of Zn flux in plants is from the soil via roots to the shoot and seeds [23]. Various transport proteins and ligands that are responsible for Zn^2+^ uptake by roots and its transport and sequestration within the plant have been characterized [12,24,25,26]. Among these, ZRT-IRT-like proteins (ZIPs) have been studied in several plants, including *Arabidopsis thaliana*, soybean (*Glycine max*), barley (*Hordeum vulgare*), barrel medic (*Medicago truncatula*), and rice (*Oryza sativa*) [27,28,29,30,31]. These proteins not only transport Zn^2+^ across membranes, but can also transport other transition metal cations, including Cd^2+^, Fe^3+^/Fe^2+^, Mn^2+^, Ni^2+^, Co^2+^, and Cu^2+^ [27,32,33]. Generally, the expression of *ZIP* genes is upregulated when plants become Zn-deficient [34,35,36], facilitating Zn influx to cells and movement of Zn between organs, and also when plants become Fe- or Mn-deficient [35,37,38,39]. Other proteins that transport Zn include the metal tolerance proteins (MTPs), which function as cation/proton antiporters and are thought to transport Zn into vacuoles [40], and the yellow stripe-like proteins (YSLs), which transport the Zn–nicotianamine complex (NA–Zn) and load Zn into the xylem and phloem [41]. The zinc-induced facilitators (ZIFs) and the heavy metal transporters (HMAs) are implicated in Zn influx to vacuoles and to the xylem, respectively [24]. Zinc is chelated by organic molecules, such as the carboxylic acid, citric acid, and nicotianamine (NA) in plants [42]. Nicotianamine is a non-proteinogenic amino acid with a high affinity for Fe, Cu, and Zn, and is involved in their homeostasis [43]. Nicotianamine mediates the intercellular and interorgan movement of Zn and was found to enable Zn hyperaccumulation in *Arabidopsis halleri* and *Noccaea caerulescens* [43,44]. In general, the functions of these transporters have been studied by expressing them in yeast, but to understand how the various Zn transport proteins and chelates act together to maintain appropriate cytosolic and tissue Zn concentrations, it is important to study the responses of an intact plant to fluctuations in Zn supply. Moreover, since there is a knowledge gap on the regulation of Zn transport following Zn foliar application, it is important also to elucidate plant transcriptional responses when Zn is not applied to roots.

**Figure 1:**
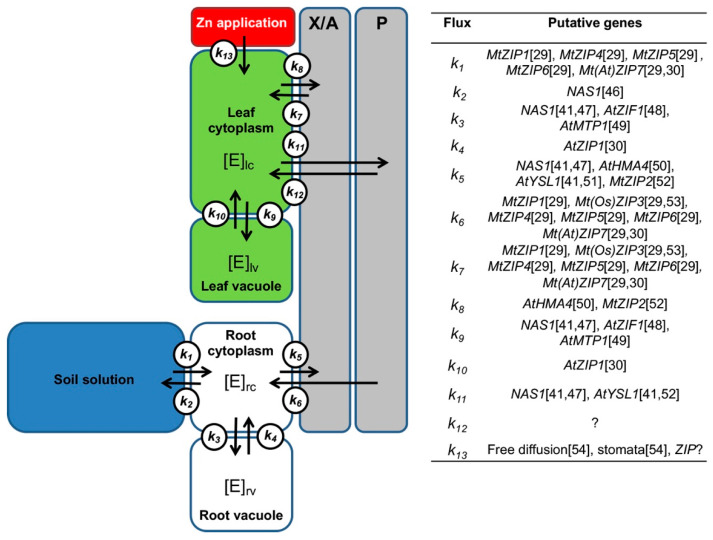
Suggested model for roles of putative genes encoding proteins involved in Zn transport- related processes. The sites of action in the plant (i.e., root cytoplasm, rc; root vacuole, rv; xylem and apoplast, X/A; phloem, P; leaf cytoplasm, lc; leaf vacuole, lv) and the element (E) fluxes (K_1–13_) are reported. The concentration of the element is indicated in each site [E]. The scheme synthetizes information across studies in various plants. Gene abbreviations: *ZIP*, Zrt-/Irt-like protein; *NAS*, nicotianamine synthase; *ZIF*, zinc-induced facilitator; *MTP*, metal transporter protein; *HMA*, P1B-type heavy metal ATPase; *YSL*, yellow stripe like protein; *ZIP*? indicates a generic *ZIP*; free diffusion: diffusion through leaf epidermis; stomata: absorption through stomata. Plant abbreviations: Mt, *Medicago truncatula*; At, *Arabidopsis thaliana*; Os, *Oryza sativa*. References: [29,30,41,46,47,48,49,50,51,52,53,54].

Thus, in this study the transcriptional responses of genes encoding Zn transport-related processes facilitating Zn uptake by cells, vacuolar sequestration, and redistribution within the plant were studied following foliar Zn application to the most productive and widely cultivated forage legume, alfalfa (*Medicago sativa* L.). The study was designed to provide information on the molecular responses to Zn biofortification of forage crops [7,45]. A model for the roles of putative genes encoding proteins involved in Zn transport- related processes was built and used for the selection of genes (Figure 1).

The following hypotheses were tested: (i) foliar application of Zn determines Zn redistribution within the plant and is associated with changes in the expression of genes involved in Zn transport-related processes; (ii) genes encoding Zn transport-related processes are organized in functional modules that act in a concerted manner to redistribute Zn within the plant to maintain non-toxic cytosolic and tissue Zn concentrations. Genes encoding putative Zn transport-related processes were identified in alfalfa through phylogenetic comparisons and their likely roles are discussed. Changes in the expression of these genes following foliar Zn application were determined and the possible effects of these on the redistribution of Zn within cells and between tissues are also discussed. The knowledge gained from this study could help to optimize Zn biofortification strategies when using foliar Zn fertilizers and to provide strategies for breeding forage crops to addresses Zn deficiencies in livestock.

## 2. Results

### 2.1. Effects of Foliar Zn Application on Plant Zn Redistribution and Expression of Genes Involved in Zn Transport-Related Processes

The first aim of this study was to provide information on Zn redistribution within the plants and on the associated transcriptional responses of Zn transport-related genes following foliar Zn biofortification to alfalfa. 

#### 2.1.1. Shoot and Root Zn Concentration and Content

**Figure 2:**
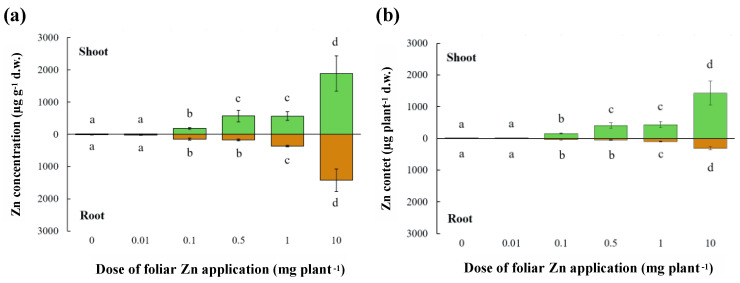
Zinc concentration (**a**) and content (**b**) in shoots and roots of alfalfa after the application of Zn to leaves. The Zn doses were 0, 0.01, 0.1, 0.5, 1, or 10 mg Zn plant^−1^. Means ± standard error of three replicates are shown. Differences among the applied Zn doses were tested separately for shoot and root by one-way analysis of variance. Different letters denote significant differences in Zn concentrations in shoots and roots independently, according to Tukey-B honestly test (*p* < 0.05).

To provide novel information on Zn redistribution within alfalfa plants following foliar Zn application, we applied six Zn doses (0, 0.01, 0.1, 0.5, 1, 10 mg Zn plant^−1^) and assessed Zn concentration and content in shoots and roots 5 days after application. The application of Zn to leaves did not modify shoot or root fresh and dry biomass (Table S1), and all *M. sativa* plants had a similar number of functional root nodules, irrespective of Zn treatments (data not shown). However, Zn concentrations in both shoots and roots were strongly affected by foliar Zn application (F_(5,17)_ = 32.61, *p*<0.001; F_(5, 17)_ = 28.53, *p*< 0.001; respectively) (Figure 2a). A foliar Zn application of 0.01 mg Zn plant^−1^ produced a shoot Zn concentration similar to that of the control (no-Zn addition), but shoot Zn concentrations were increased progressively by larger doses (0.1 < 0.5/1 < 10 mg Zn plant^−1^), from more than 3-fold to 35-fold more than that of the control (Figure 2). Foliar applications of 0.01, 0.1, and 0.5 mg Zn plant^−1^ did not produce root Zn concentrations greater than that of the control treatment, but foliar doses of 1 and 10 mg Zn plant^−1^ increased root Zn concentrations to 3-fold and 11-fold more than the control treatment, respectively. Shoot and root Zn contents were also strongly affected by foliar Zn application (F_(5,17)_=53.73, *p* < 0.001; F_(5, 17)_ = 32.45, *p* < 0.001; respectively) and their responses to increasing foliar Zn applications followed the corresponding Zn concentrations (Figure 2b). At all Zn dose plants did not show any visual symptom of Zn deficiency or toxicity. Moreover, plants grown for two months lacking Zn (i.e., 0 mg Zn plant^−1^) had shoot and root Zn content of 4.5 and 2.7 µg plant^−1^, respectively, probably relying on seed Zn content.

To summarize, the efficacy of Zn biofortification (i.e., shoot Zn concentrations in the range 15–400 μg Zn g^−1^ d.w.) was proved for the doses of 0.1 to 1 mg plant^−1^, while the lowest dose (0.01 mg plant^−1^) was ineffective, and the highest dose (10 mg plant^−1^) produced toxic concentrations.

#### 2.1.2. Phylogenetic Analysis

To infer the putative roles of the selected *M. sativa* Zn transport-related genes, we performed phylogenetic analyses. This was based on the assumption of a simple equivalence between a minimum similarity threshold in the phylogenetic comparisons and the function similarity between encoded proteins. Phylogenetic analysis of the coding sequences of the *ZIP* genes revealed several distinct clades (Figure S1). One clade contained sequences for *MsZIP2* and *MsZIP7*, which were similar to each other. In addition, the sequence of *MsZIP2* was closely related to those of *MtZIP2* and *GmZIP1*-*ZIP2*, and the sequence of *MsZIP7* was closely related to those of *MtZIP7* and *AtZIP11*. Another clade contained the sequences of *MsZIP1*, *MsZIP3*, *MsZIP5*, and *MsZIP6*. The sequence of *MsZIP1* clustered with that of *MtZIP1*. Sequences of *MsZIP3* and *MsZIP5* were similar to each other and clustered with the corresponding sequences for *M. truncatula* genes (Figure S1). Sequences for *MsZIP1*, *MsZIP3*, and *MsZIP5* were closely related to each other, whereas that of *MsZIP6* formed a separate cluster with the sequences of *MtZIP6* and *AtZIP12*. The sequence of *MsZIP4* was distant from the sequences of other *M. sativa* ZIPs and formed a cluster with the sequences of *MtZIP4* and *AtZIP4*.

Phylogenetic analyses of the coding sequences of the other genes related to Zn transport processes revealed that they were all similar to their *M. truncatula* counterparts. As regards *ZIF*, the sequence of *MsZIF1* clustered with the sequences of *MtZIF1* and *GmZIF1* (Figure S2a). As regards *MTP*, the sequence of *MsMTP1* formed a cluster with *MtMTP1* and *GmMTP1* and was also related to *AtMTP1* and *AtMTPA1* (Figure S2b). Similarly, the sequence of *MsYSL1* was most similar to those of *MtYSL1* and *GmYSL1* (Figure S3a) and the sequence of *MsHMA4* was most similar to those of *MtHMA4* and *GmHMA4* (Figure S3b). Finally, the sequence of *MsNAS1* was closely related to those of *MtNAS* and *GmNAS* (Figure S4).

To summarize, the genes selected for gene expression analysis were closely related to the homologous of *M. truncatula* and of other plant species. Thus, on the basis of the pattern of clustering and of the functions described in literature for the encoded proteins, we were able to infer the putative roles of the genes.

#### 2.1.3. Gene Expression Analysis

**Figure 3:**
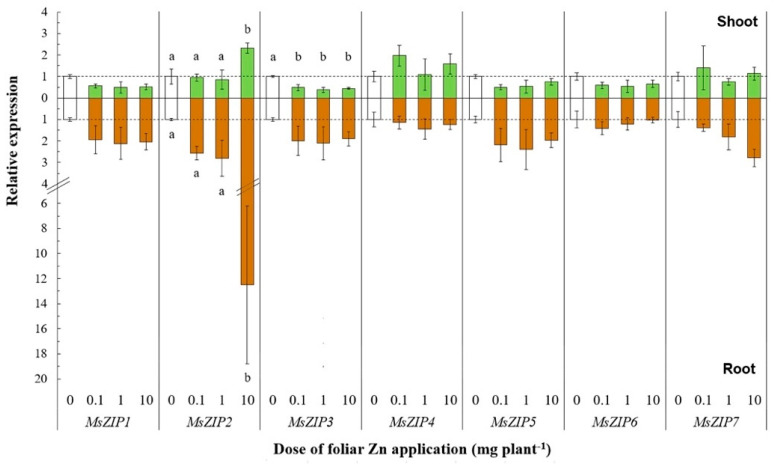
Relative expression of transmembrane Zn transporter genes after leaf Zn application to alfalfa. The Zn doses were 0, 0.1, 1, or 10 mg Zn plant^−1^. Means ± standard error of three replicates are shown. The expression levels were calculated relative to reference genes (*MsACT-101* for shoot and *MsEF1-α* for root) and to the control (0 mg Zn plant^−1^). The broken line denotes the threshold between up- and downregulation relative to the control. Differences in the expressions of each gene after different Zn doses were tested separately for shoot and root by one-way analysis of variance. Different letters denote significant differences among Zn doses, according to Tukey-B test (*p* < 0.05).

To provide novel information on the transcriptional responses of genes encoding Zn transport-related processes following foliar Zn application, we analyzed the expression of *MsZIP1-7*, *MsMTP1*, *MsYSL1*, *MsHMA4*, and *MsNAS1* genes in shoots and roots, 5 days after Zn application of 0, 0.1, 1, and 10 mg Zn plant^−1^. The Zn treatments were selected on the basis of the significance of the results on Zn redistribution in shoots and roots. The expression of *MsZIP3* was significantly downregulated only in shoots at foliar doses of 0.1, 1, and 10 mg Zn plant^−1^ (F_(3, 11)_ = 28.46, *p* < 0.01) (Figure 3). By contrast, the expression of *MsZIP2* was significantly upregulated in shoots and roots at the largest dose of 10 mg Zn plant^−1^ (F_(3, 11)_ = 5.59, *p* < 0.05; F_(3, 11)_ = 9.26, *p* < 0.01). The expression of *MsZIP1*, *MsZIP5*, and *MsZIP6* in shoots was not significantly affected by foliar Zn application, although a general trend towards downregulation with increasing foliar Zn doses was observed. The expression of *MsZIP4* and *MsZIP7* in shoots was unaffected by foliar Zn application. 

**Figure 4:**
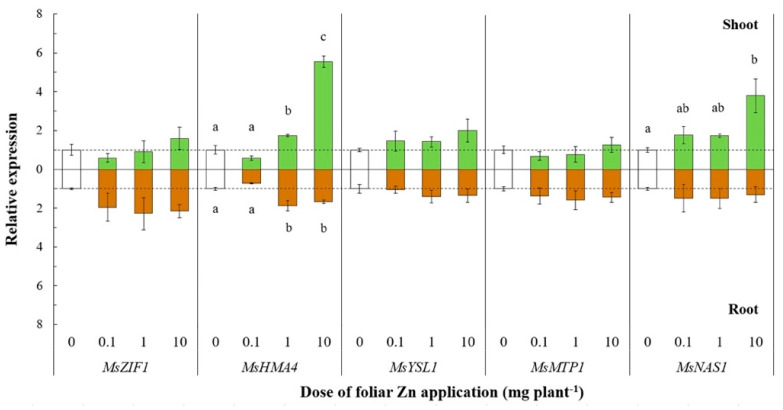
Relative expression of genes related to Zn transport processes after leaf Zn application to alfalfa. The Zn doses were 0, 0.1, 1, or 10 mg Zn plant^−1^. Means ± standard error of three replicates are shown. The expression levels were calculated relative to reference genes (*MsACT-101* for shoot and *MsEF1-α* for root) and to the control (0 mg Zn plant^−1^). The broken line denotes the threshold between up- and downregulation relative to the control. Differences in the expression of each gene at the different Zn doses were tested separately for shoot and root by one-way analysis of variance. Different letters denote significant differences among Zn doses, according to Tukey-B test (*p*< 0.05).

In roots, all *ZIP* genes except *MsZIP2* were not significantly affected by foliar Zn application, although a general trend of *MsZIP1*, *MsZIP3*, *MsZIP5*, and *MsZIP7* towards upregulation with increasing foliar Zn doses was observed. Of the other genes related to Zn transport processes, the expression of *MsHMA4* was significantly upregulated in both shoots (F_(3, 11)_ = 115.29, *p* < 0.01) and roots (F_(3, 11)_ = 14.23, *p* < 0.01) following the application of 1 and 10 mg Zn plant^−1^ (shoots: +63% and +424%, respectively; roots: +86% and +66%, respectively; Figure 4).

In shoots, the expression of *MsHMA4* was about threefold higher following a dose of 10 mg Zn plant^−1^ than following a dose of 1 mg Zn plant^−1^, whereas the expression of MsHMA4 in roots was similar when 1 or 10 mg Zn plant^−1^ was applied.

The expression of *MsNAS1* was also significantly upregulated (F_(3, 11)_ = 6.46, *p* < 0.05) at the largest foliar Zn dose (10 mg plant^−1^), whereas its expression in roots was unaltered following foliar Zn application (Figure 4). In shoots, *MsYSL1* and *MsZIF1* were not significantly affected by foliar Zn application, although there was a trend towards upregulation of the expression with increasing Zn doses, while the expression of *MsMTP1* remained unaltered following the application of Zn (Figure 4). Finally, in roots, *MsMTP1* and *MsZIF1* were not significantly affected by foliar Zn application, although there was a trend towards upregulation of the expression with increasing Zn doses, whereas the expression of *MsYSL1* remained unchanged (Figure 4).

The permutation analysis of variance (PERMANOVA) showed that the expression of *ZIP* genes was significantly affected by foliar Zn application dose and differed between shoots and roots, which explained 29% and 23% of the total variance, respectively (Table 1). The expression of other genes related to Zn transport processes that were studied (*MsZIF1*, *MsNAS1*, *MsHMA4*, *MsYSL1*, and *MsMTP1*) were also affected by foliar Zn application dose and the organ examined. Zinc application dose explained 17% of the total variance, while plant organ explained 19%. PERMANOVA on all studied genes highlighted a significant effect of Zn application dose, plant organ, and their interaction on gene expression, explaining 68% of the total variance.

To summarize, among the 12 studied genes, only the expression of *MsZIP2, MsZIP3, MsHMA4*, and *MsNAS1* changed after foliar Zn application. *MsZIP2* and *MsHMA4* were upregulated in shoots and roots, whereas *MsZIP3* was downregulated and *MsNAS1* upregulated only in shoots.

### 2.2. Functional Modules of Genes Encoding Zn Transport-Related Processes

**Figure 5:**
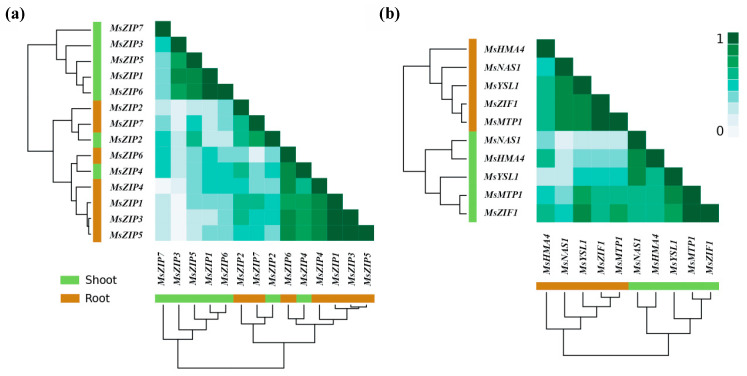
Heatmaps reporting correlations between expression of genes related to Zn transport after foliar Zn application. Gene expression is calculated as the difference between 0.1, 1, or 10 mg Zn plant^−1^ and a control of 0 mg Zn plant^−1^. The similarity in the degree of correlation in fold-change of gene expression to Zn application relative to the control was based on the average linkage clustering of the Pearson correlations (*r*). In the clustering trees, the genes are indicated in brown for roots and in green for shoots, while the ranks of correlations of the heatmap are indicated by color intensity (*r* 0 to 1: from low to strong intensity of green). Seven genes encoding transmembrane Zn transporter (*MsZIP1-7*) (**a**); four genes encoding cellular Zn transporters (including vacuolar transporters) (*MsZIF1*, *MsHMA4*, *MsYSL1*, and *MsMTP1*) and a gene encoding a nicotianamine synthase (*MsNAS1*) (**b**).

The second aim of this study was to provide novel information on how Zn transport-related genes are organized in functional modules in alfalfa. Using correlation analysis to reveal functional modules of genes whose expression is co-regulated in plants, we observed three functional modules for co-expression (Figure 5; *r* > 0.6). The first functional module of genes consisted of *MsZIP1*, *MsZIP5*, and *MsZIP6* in shoots and of *MsZIP1*, *MsZIP3*, *MsZIP4*, *MsZIP5*, and *MsZIP6* in roots. The second functional module of genes consisted of *MsMTP1* and *MsZIF1* in both shoots and roots. The third functional module of genes consisted of *MsHMA4*, *MsYSL1*, and *MsNAS1* in both shoots and roots. Moreover, while the expression pattern of ZIPs in shoots did not diverge from the one in roots, the expression pattern of the other genes involved in Zn transport-related processes strongly diverged (Figure 5).

The identification of these modules may allow for the definition of how the genes act in a concerted manner to redistribute Zn within the plant, maintaining non-toxic cytosolic and tissue Zn concentrations.

## 3. Discussion

In this work, for the first time, Zn biofortification was applied to the most productive and widely cultivated forage legume, alfalfa. Specific qPCR assays were designed for this crop and were successfully validated to study the gene expression response to foliar Zn application. We firstly characterized the expression of Zn transport-related genes after foliar Zn application to alfalfa and provide new molecular insights by identifying three functional gene modules involved in Zn influx to cells, Zn sequestration in the vacuole, and Zn redistribution within the plant.

### 3.1. Zn Redistribution within the Plant after Foliar Zn Application 

The critical leaf concentration for Zn deficiency approximates 15–20 μg Zn g^−1^ dry weight and the critical leaf concentration for Zn toxicity approximates 400–500 μg Zn g^−1^ [12,13]. Before foliar Zn application, the alfalfa plants used in the experiments reported here were probably Zn-deficient, since their shoot Zn concentrations were below the critical leaf concentration for Zn deficiency (Figure 2). After the application of the lowest foliar Zn dose (0.01 mg plant^−1^), plants probably remained Zn-deficient (7.6 μg Zn g^−1^ dry weight), but all other foliar Zn doses increased Zn concentrations in shoots above the critical concentration for Zn deficiency (Figure 2). Plants treated with 0.1 mg Zn plant^−1^ probably had an optimal Zn status for plant growth, whereas plants treated with 0.5 and 1 mg Zn plant^−1^ had shoot Zn concentrations close to the toxicity threshold. When a foliar dose of 10 mg Zn plant^−1^ was applied, shoot Zn concentrations greatly exceeding the threshold for Zn toxicity (Figure 2). Plants often exhibit characteristic visual symptoms of Zn deficiency and Zn toxicity when these occur [12,13], but 5 days after foliar Zn application, no visual symptoms of Zn deficiency or toxicity, nor differences in plant biomass, were observed among plants receiving contrasting foliar Zn doses (data not shown). Foliar Zn doses larger than 0.1 mg Zn plant^−1^ resulted in incremental increases in the Zn concentration and content of roots (Figure 2), despite Zn having limited mobility in the phloem [20,55]. This observation suggests that roots can act as a sink for Zn applied to leaves, thereby mitigating excessive Zn accumulation in shoot tissues.

In previous work, foliar application of Zn was shown to increase Zn concentration in phloem-fed tissues, such as fruits, seed, and tubers [56,57,58,59]. The shoot to root Zn concentration ratio shifted from values below one in conditions of Zn deficiency (0.4) to values greater than one in Zn-replete or Zn-intoxicated plants (1.3–3.2) (Figure 2). When the plants are Zn-deficient, the recirculation of Zn between organs via the xylem and phloem is required to meet minimal growth demands and the application of foliar Zn to Zn-deficient plants must be effectively redistributed within the plant [42,60], whereas when excessive foliar Zn is applied, Zn must be chelated in the cytoplasm, sequestered in the vacuole, and redistributed via the phloem or xylem to other organs to avoid toxicity [12].

### 3.2. Phylogenetic and Gene Expression Analysis

Despite several genes encoding Zn transporters having been identified in plants, and the encoded proteins characterized, the mechanisms of Zn uptake and transport in alfalfa are still largely unknown. However, the recently sequenced alfalfa genome has allowed for the discovery of genes involved in Zn uptake and distribution within this species [61]. 

The influx and efflux of Zn across the plasma membrane of plant cells must be tightly controlled to allow optimal cell functioning and hence to ensure normal plant growth and development [42]. The expression of only two of the seven *ZIP* genes studied, *MsZIP2* and *MsZIP3*, showed statistically significant responses to foliar Zn application (Figure 3). The expression of *MsZIP2* was significantly upregulated in both shoots and roots in response to the largest dose of foliar Zn applied (10 mg Zn plant^−1^). It is likely that this dose is toxic to both shoot and root cells. The relative induction in the expression of *MsZIP2* was greatest in roots. The phylogenetic analysis of *ZIP* transporters revealed that *MsZIP2* is closely related to *MtZIP2* and *AtZIP2* (Figure S1). The *AtZIP2* protein was previous found in the same clade with HsZIP2 [62]. Thus, *MsZIP2* is probably located in the plasma membrane performing similar functions to *MtZIP2*, *AtZIP2*, and *HsZIP2*. The authors of [52] reported that *M. truncatula* plants grown with adequate soil Zn availability expressed *MtZIP2* in roots and stems, but not in leaves. The expression of *MtZIP2* in roots increased with increasing Zn fertilizer applications to soil, with the greatest expression being found at toxic Zn doses [52]. Similarly, the authors of [30] found that the expression of *AtZIP2* was ≈10-fold higher in roots than shoots in Zn-replete *Arabidopsis thaliana* plants and that Zn deficiency reduced the expression of *AtZIP2* in both roots and shoots. The localization of *ZIP2* at the plasma membrane was observed in both *M. truncatula* [49] and *A. thaliana* [30]. The expression of *AtZIP2* was localized to the stele of the root [30], supporting a role of *AtZIP2* in long distance transport of Zn between roots and shoots. It is possible that the increased expression of *MsZIP2* observed in our study when plants experience Zn toxicity might be a detoxification strategy, either through storing excess Zn in xylem parenchyma cells or recirculating Zn in the xylem.

The expression of *MsZIP3* was significantly downregulated in shoots following the foliar application of Zn (Figure 3). The *ZIP3* transporter is thought to mediate Zn influx to the cell from the apoplast [42]. Therefore, the downregulation of *MsZIP3* in shoots of plants receiving more Zn is consistent with the ability of plant cells to control their Zn uptake to affect cytoplasmic Zn homeostasis. Reduced expression of *MsZIP3* in plants with a greater Zn supply is also in agreement with previous studies of *M. truncatula* and *A. thaliana* [27,29], despite the higher phylogenetic similarity of *MsZIP3* to *MtZIP3* than to *AtZIP3* (Figure S1). However, although *AtZIP3* could restore growth to a Zn uptake-defective yeast [30], *MtZIP3* was not found to be able to restore the growth of a Zn uptake-defective yeast in Zn-limited media, although it did restore the growth of a Fe uptake-defective yeast in Fe-limited media [29]. Thus, the MsZIP3 transporter could have a higher affinity for Fe than Zn. In *O. sativa*, the *ZIP3* gene is expressed in the xylem parenchyma and transfer cells and might be responsible for unloading transition metal cations from the xylem to the parenchyma in plants receiving an excessive Zn supply [53]. The role of OsZIP3 in unloading Zn from the vascular tissues suggests that the reduced expression of *MsZIP3* in shoots of *M. sativa* receiving an excessive foliar Zn dose might be a detoxification strategy to reduce Zn uptake by shoot cells.

The observation that foliar Zn applications had no effect on the expression of *ZIP* genes, except *MsZIP2* and *MsZIP3* (Figure 3), might be explained by the roles of ZIP proteins in the transport of other transition metals. For example, evidence of Cu and Mn transport by *ZIP4* were provided through yeast complementation studies [29,63]. Moreover, applying the same technique, a role of *ZIP6* was highlighted in the transport of Fe by [29], whereas the authors of [63] did not find any involvement of *ZIP6* in the transport of Cu, Zn, or Fe. Although the changes in the expression of *MsZIP1*, *MsZIP5*, and *MsZIP6* following foliar Zn application were not statistically significant, changes in their expression in shoots were positively correlated with changes in the expression of *MsZIP3*, showing a general trend for them to be downregulated following foliar Zn application and suggesting that these four ZIPs might act as a functional module in the shoot (Figure 5). By contrast, the expression of *MsZIP1*, *MsZIP3*, *MSZIP4*, and *MsZIP5* were positively correlated in roots, suggesting that these genes behave as a functional module in roots.

The expression of *MsHMA4*, which is implicated in Zn redistribution within the plant [50,51,52,53,54,55,56,57,58,59,60,61,62,63,64], was increased in both shoots and roots of plants whose shoot Zn concentration suggested they were close to, or experiencing, Zn toxicity (Figure 4). The significant upregulation of *MsHMA4* following foliar application of ≥ 1 mg Zn plant^−1^ might be related to the removal of excess Zn from both shoots and roots. This interpretation is consistent with the role of HMA4 in *A. thaliana* and in the metal hyperaccumulators *Arabidopsis halleri* and *Noccea caerulescens* [12,50,65,66,67], in which greater expression of *HMA4* results in greater Zn flux to the xylem and Zn translocation to transpiring leaves. However, the phylogenetic similarity of *MsHMA4* to *MtHMA4* and, particularly, to *AtHMA5* (Figure S3b) suggests a role in Cu transport [68,69,70]. The implication of the latter observation is unclear.

Since Zn^2+^ concentrations are low in the alkaline phloem sap, the transport of most Zn in the phloem is as Zn ligand complexes, such as zinc–nicotianamine (NA–Zn) [71]. Nicotianamine is the main Zn chelate in phloem transport and is also important for Zn sequestration in vacuoles [43], and tolerance of excessive Zn uptake [46]. Nicotianamine concentrations generally correlate with those of *NAS* transcripts, and for this reason *NAS* expression can be used as a proxy for NA content [48,72]. Accordingly, in the work reported here the increased expression of *MsNAS1* in shoots following the application of ≥ 1 mg Zn plant^−1^ (Figure 4) probably reflects the role of NA in Zn detoxification through its sequestration within vacuoles and its redistribution from shoot to root after excessive foliar Zn applications. This observation is consistent with the work of [71], who found that the expression of *NAS2* in wheat increased following foliar Zn application, despite the high phylogenetic distance of the *NAS* genes in *M. sativa* and wheat (Figure S4). Moreover, other authors reported that *NAS* expression is constitutively high in plants that hyperaccumulate Zn [12,72,73,74].

Homologs of *MsMTP1* and *MsZIF1* were previously found to encode transporters loading Zn and NA into the vacuoles of *Thlaspi geosingense* and *A. thaliana* cells, respectively [48,75]. Unexpectedly, the expression of these genes was unaffected by foliar Zn application (Figure 4). This observation suggests that the proteins encoded by these genes might not contribute to Zn detoxification in *M. sativa*. Nevertheless, only *MsZIF1* of all the genes studied here showed a trend towards increased expression in roots with increasing foliar Zn dose, which might indicate a role in detoxification of excess Zn in roots through its sequestration with NA in the vacuole. 

In *A. thaliana*, *AtYSL1* has a role in the long-distance transport of the NA–Zn complex and in loading Zn into seeds [41,76]. For this reason and according to the similarities in the phylogenetic tree between *MsYSL1/MtYSL1* and *AtYSL1* (Figure S3a), an increase in the expression of *MtYSL1* was expected to occur in parallel with the increased expression of *MsNAS1* in shoots. However, the expression of *MsYSL1* did not show any significant change in shoots or roots in response to foliar Zn application, although there was a trend towards greater *MsYSL1* expression in shoots with increasing foliar Zn doses (Figure 4). 

### 3.3. Functional Modules of Genes Encoding Zn Transport-Related Processes

The responses of gene expression to foliar Zn applications suggest three functional modules that effect cytoplasmic Zn homeostasis through Zn transport-related processes in *M. sativa*: genes involved in Zn influx to cells (shoots: *MsZIP1*, *MsZIP5*, and *MsZIP6*; roots: *MsZIP1*, *MsZIP3*, *MSZIP4*, *MsZIP5*, and *MsZIP6*), genes involved in Zn sequestration in the vacuole (shoots and roots: *MsMTP1* and *MsZIF1*), and genes involved in Zn redistribution within the plant (shoots and roots: *MsHMA4*, *MsYSL1*, and *MsNAS1*) (Figure 5). In a previous work that jointly analyzed the structures and phylogenetic trees of 21 *ZIP* genes in *Populus trichocarpa* in response to metal stress, four classes of genes were identified [77]. Among them, class I and class II were identified as involved in the transportation and absorption of metal ions (i.e., Zn, iron, copper, and manganese) during nutritional surpluses, while class III and class IV were identified as induced for metal ion transport during stress. Similarly to our results, in *PtrZIP1*, *PtrZIP4*, *PtrZIP5*, and *PtrZIP6* belonged to the same class (i.e., class I), but the pattern of gene expression under Zn deficiency and Zn application differed. Accordingly, a joint sequence and expression analysis of ZIP transporter genes revealed coexpression networks in iron acquisition strategies in land plants as well as in green algae [78].

The high correlation found in the present work between the expression of *MsMTP1* and *MsZIF1* in both shoots and roots (Figure 5) is also supported by previous works reporting a synergistic action of these gene for the sequestration of Zn in the vacuole [48,75,79]. Finally, the high correlation in the expression of *MsHMA4*, *MsYSL1*, and *MsNAS1* found in shoots and roots in response to foliar Zn applications (Figure 5) supports the expectation that these genes are components of a functional module affecting the long-distance transport of Zn in the plant, as it was previously highlighted in *A. thaliana* by [80].

However, more effort should be made in further studies to verify the localization of those proteins within cell and tissue of *M. sativa* as well as of other known orthologs involved in Zn transport. Moreover, additional time course studies should be performed to account for time-dependent responses.

## 4. Materials and Methods

### 4.1. Plant Growth and Experimental Design

Surface-sterilized seeds of alfalfa (*M. sativa* L.) were germinated on moist sterilized silica sand (1–4 mm size) in a climatic chamber at 24/21 °C day/night temperature, 16/18 h light/dark cycle, and 200 µmol photons m^−2^ s^−1^. After 2 weeks of growth, 3 seedlings were transplanted to 1500 mL volume pots and filled with sterilized silica sand (number of pots 18), and *Sinorhizobium meliloti* was supplied as a filtrate to all plants to ensure that the plants produced nodules in all treatments. A Hoagland nutrient solution lacking Zn [81] was used to fertilize the plants, with 10 mL solution being applied every week. After 2 months of growth, when plants were in the vegetative growth stage, plants were treated with 1 of 6 doses of Zn (0, 0.01, 0.1, 0.5, 1, and 10 mg Zn plant^−1^) (3 replicates per dose). Six ZnSO_4_·7H_2_O solutions of 0, 0.05, 0.5, 2.5, 5, and 50 g Zn L^−1^ were prepared to supply these doses. A drop of Tween 20 detergent was added to the 6 solutions to break the surface tension of the leaves and enhance Zn uptake. Zinc was applied to the middle leaf laminae of the 3 plants in each pot as twenty 10 μL droplets. The experiment was arranged in a fully randomized design, with 3 replicates for each Zn dose. The shoots and roots of the plants were harvested separately 5 days after Zn application. At harvest, 1 mM CaCl_2_ solution and water were used to remove any residual Zn from the leaf surface [82]. Shoot and root fresh weight was measured, whereas shoot and root dry weight was determined on subsamples after oven drying at 70 °C to constant weight.

### 4.2. Measurement of Zn Concentration

Approximately 100 mg of shoot or root dry biomass was carefully weighed and mineralized in a microwave medium pressure digestor (Milestone Start D, FKV Srl, Torre Boldone, Italy) with 7 mL of 69% HNO_3_ and 2 mL of 30% H_2_O_2_ (ultrapure grade). Zinc concentration in the resulting solutions was determined by inductively coupled plasma optical emission spectroscopy (ICP-OES) using an Optima 8000 spectrometer (Perkin Elmer, Waltham, MA, USA), following the procedure of [83].

### 4.3. Gene Selection and Design and Validation of New RT-qPCR Assay

Seven genes encoding putative ZRT-IRT-like proteins (ZIP) were selected for investigation (i.e., *ZIP1-7*) (Figure 1). The selection was based on information gathered by [29,52] on the expression of genes encoding Zn transporters in the model legume *M. truncatula* and on the structure of the neighbor-joining (NJ) tree built using available ZIP sequences of several plant species. Five more genes, whose products are involved in Zn transport-related processes [24], were also chosen for investigation on the basis of information gathered by other authors and on sequence similarity with other plant species. The *NAS1* gene encoding nicotianamine synthase (NAS) was chosen because this enzyme synthesizes nicotianamine (NA), which is involved in long-distance Zn transport [41] (Figure 1). The *HMA4* gene, which encodes a transmembrane P-type ATPase heavy metal transporter, was chosen because this transporter loads Zn into the xylem in roots for its transport to shoots [51] (Figure 1). The *MTP1* gene, which encodes a transporter of the CDF family, was selected because this transporter is implicated in the sequestration of excess Zn in the vacuole [49,75] (Figure 1). The *ZIF1* gene, which encodes the Zn-induced facilitator 1 transporter, was chosen because it transports NA into the vacuole to chelate vacuolar Zn [48,84] (Figure 1). The *YSL1* gene, which encodes a transporter of NA–Zn complexes, was chosen because it is implicated in Zn loading and transport of Zn in the phloem [60] (Figure 1). To standardize the expression of genes encoding Zn transport-related processes, we selected 2 reference genes: actin (*ACT-101*) and elongation factor 1-α (*EF1-α*) [85].

Using the draft genome sequence of alfalfa in the Alfalfa Gene Index and Expression Atlas Database (AEGD) [61] (http://plantgrn.noble.org/AGED/index.jsp), we retrieved homologous gene sequences of *M. sativa* by Basic Local Alignment Search Tool (BLAST) similarity searches using the gene sequences of *M. truncatula*. The chosen genes for *M. sativa* were named *MsZIP1-7* for the seven *ZIP* genes and *MsNAS1*, *MsHMA4*, *MsZIF1*, *MsYSL1*, and *MsMTP1* for the other selected genes. The two reference genes were named *MsACT-101* and *MsEF1-α*. The gene sequences and their annotations have been deposited in the National Center for Biotechnology Information (NCBI) under the submission # 2338923.

Forward and reverse new PCR primers for the 12 Zn transport-related genes and the 2 reference genes suitable for SYBR Green II RT-qPCR assays (Biorad, USA) were designed (Table 2). The Primer-BLAST online tool in the National Center for Biotechnology Information (NCBI; https://www.ncbi.nlm.nih.gov/tools/primer-blast/ (accessed on 2 March 2021)) was used to design primers. The newly designed RT-qPCR assays are suitable for both *M. sativa* and *M. truncatula*. These assays are the first designed specifically for alfalfa and resulted to be more efficient than the ones already available for *M. truncatula* (i.e., *MtZIP1-7* and *MtMTP1;* [29,52,86]). The length of the fragment, the Sanger sequences of the PCR amplicons (Table 1), and the single melting temperature peaks confirmed the specificity of the new RT-qPCR assays (Figure S5). Sanger sequencing was performed on PCR amplicons of 3 complementary DNA (cDNA) samples (Material and Methods S1). Examples of electropherograms of the sequences are reported in Figures S6 and S7. The sequences of the obtained PCR amplicons have been deposited in the NCBI under the submission # 2338930. Amplification efficiencies (E) in the range of 96.1–111.0% were evidence of accurate quantification, while the coefficients of correlation (*R^2^* > 0.998) indicated a high precision of measurements across concentration ranges of at least 3–4 orders of magnitude (Table 2 and Figure S8). The concentration ranges over which the relationship between the relative fluorescence and the logarithm of the concentration was linear, and the precision of quantification (standard curves) as reflected in the coefficient of correlation (*R^2^*), was determined using 3 independent 10-fold serial dilutions of a cDNA sample of *M. sativa*. The accuracy of quantification was determined by the efficiency (E) of each qPCR amplification using the equation E = [10^−1/S^ − 1] × 100, where S is the slope of the standard curve. The evaluation of the reference genes based on the cycle threshold (Ct) values made us choose the actin gene (*MsACT-101*) for quantifying relative gene expression in the shoots and the elongation factor 1-*α* (*MsEF1-α*) gene for quantifying relative gene expression in roots (Figure S9a,b). This choice was based on the observations that there was no statistical difference in the expression of the reference genes in tissues following foliar Zn applications and that *MsACT-101* and *MsEF1-α* showed the smallest overall variation in the shoot and root, respectively (Figure S9c,d).

### 4.4. RNA Extraction and Gene Expression Analysis

Total RNA was extracted from 50 mg subsamples of fresh shoot and root tissue using the RNeasy Mini Kit (Qiagen, Hilden, Germany). The extractions were performed from tissues of plants treated with the foliar Zn doses that produced a significant increase in Zn concentration in shoots (0.1, 1, and 10 mg Zn plant^−1^) and the control plants to which no foliar Zn had been applied (24 RNA extractions). Any DNA in the RNA extracts was removed by a DNase treatment (Promega, USA). The purity of the RNA extracts was verified by spectroscopic light absorbance measurements at 230, 260, and 280 nm using the NanoDrop 2000 (Thermo Scientific, Worcester, MA, USA) [87]. The integrity and approximate concentration of the extracted RNA was determined by electrophoresis of the RNA extracts in a 1% agarose gel containing Sybr Safe (Invitrogen, Carlsbad, CA). One microgram of total RNA was reverse transcribed to complementary DNA (cDNA) using the iScript cDNA Synthesis Kit (Biorad, Hercules, California) in a 20 μL reaction volume. The RT-qPCRs for gene expression analysis were run as 3 technical replicates with a final reaction volume of 20 μL, containing 10 μL of SYBR Green Supermix (Biorad, Hercules, California), 5 μL of 100-fold diluted cDNA, and 0.4 μM final concentrations of the gene-specific PCR primers on a CFX Connect Real-Time System thermal cycler (Biorad, Hercules, California). The qPCR conditions were 95 °C for 3′, followed by 40 cycles of 95 °C for 5’, and 60 °C for 30″. A dissociation curve of each reaction was performed (65° C to 95° C, 0.5° C increment every 5″) to check that PCR amplified only one product. The most suitable reference gene for relative gene expression analysis was determined by comparing the expression levels of the reference genes *MsACT-101* and *MsEF1-α* across all cDNA samples. Relative gene expression was calculated using the double standardization (ΔΔCq) method that requires a reference gene and a control treatment [88].

### 4.5. Bioinformatic and Statistical Analyses

A BLAST search was performed in the Alfalfa Gene Index and Expression Atlas database using the ZIP1-7, ZIF1, MTP1, YSL1, HMA4, and NAS coding sequences from *M. truncatula*. This allowed for the identification of gene sequences encoding potential metal transporters and chelators in the whole *M. sativa* genome. The sequences obtained were aligned with the corresponding sequences from *M. truncatula*, and the length of the *M. sativa* genes was determined after removing the external unaligned nucleotides. The *M. sativa* and *M. truncatula* ZIP gene sequences were also aligned with those of other plant species (*A. thaliana*, *G. max*, *H. vulgare*, *O. sativa*, *Triticum aestivum*, and *Zea mays*) obtained from a search of GenBank. Similarly, the *M. sativa* and *M. truncatula* gene sequences of ZIF1, MTP1, YSL1, HMA4, and NAS were aligned with their corresponding sequences of other plant species (*A. thaliana*, *G. max*, *H. vulgare*, *O. sativa*, *T. aestivum*, and *Z. mays*) obtained from a search of GenBank. Sequence alignments were performed using the algorithm ClustalW in MEGA X [89]. Phylogenetic comparisons were performed to infer the putative roles of the selected *M. sativa* Zn transport-related gens. This was based on the assumption of a simple equivalence between a minimum similarity threshold in the phylogenetic comparisons and the function similarity between encoded proteins. For some proteins belonging to the same family, this assumption can hold true, since they have been shown to have very tightly correlating functions, such as those considered in this study. Thus, functions are indicated with high probability by annotations based on similarities. The phylogenetic trees were inferred by neighbor-joining (NJ) analysis [90] in MEGA X, and the evolutionary distances were calculated using the p-distance method [91]. Branch support bootstrap values were derived from 500 bootstrap replicates. The phylograms were drawn by MEGA X and edited using Adobe Illustrator CC 2017.

The effect of the application of the foliar Zn on tissue Zn concentration and on the expression of the selected genes was analyzed in shoots and roots separately by one-way analysis of variance (ANOVA), followed by a Tukey-B test in the case of significance of the response to foliar Zn application. When required, gene expression data were log-transformed to meet the ANOVA assumptions. The data displayed graphically are the means and associated standard errors of the untransformed raw data. All statistical analyses were performed using the software package SPSS version 21.0 (SPSS Inc., Chicago, IL, USA). Permutational analysis of variance (PERMANOVA) [92] was used to test the effect of foliar Zn application and plant organ (shoot and root) on the expression of the 7 ZIP genes and of the other 5 genes encoding Zn transport-related processes separately. In addition, the PERMANOVA was performed on the expression of all the genes together. The response data matrices were standardized by sample and total, and then Euclidean distances were calculated among samples. *P*-values were calculated using the Monte Carlo test [93]. Since PERMANOVA is sensitive to differences in multivariate location and dispersion, analysis of homogeneity of multivariate dispersion (PERMDISP [94]) was performed to check the homogeneity of dispersion among groups. The analyses were performed using PRIMER 7 and PERMANOVA+ software [95]. Finally, heatmaps were constructed to illustrate correlations in expression among ZIPs and among other genes encoding Zn transport-related processes using the R package ggplot2 [96], using the average linkage clustering of the Pearson correlations calculated from relative gene expression following foliar Zn application.

## 5. Conclusions

This is the first study to characterize the expression of genes related to Zn transport processes following foliar Zn application to a forage legume, providing new molecular insights to the responses of Zn transport-related processes to foliar Zn applications. A significant increase in the expression of *MsZIP2* as foliar Zn doses increased suggests the detoxification of excess Zn through the accumulation of Zn in xylem parenchyma cells. A decrease in the expression of *MsZIP3* as foliar Zn doses increased suggests a reduction in the Zn influx capacity of shoot cells to reduce Zn uptake. An increase in the expression of *MsHMA4* in roots and shoots as foliar Zn doses increased suggests an increase in the transport of Zn in the xylem when plants are subject to Zn toxicity, while an increase in the expression of *MsNAS1* in the shoot suggests the chelation of excess Zn in the shoot, enabling Zn sequestration in vacuoles or the redistribution of Zn to roots via the phloem. The elucidation of three functional modules of genes involved in (a) Zn influx to cells, (b) sequestration of Zn in the vacuole, and (c) redistribution of Zn within the plant are fundamental to understanding the molecular mechanisms of cytoplasmic Zn homeostasis and might inform the selection of appropriate genotypes enabling greater Zn accumulation in edible portions or increased tolerance of Zn in the environment.

## Figures and Tables

**Table 1:** Permutation analyses of variance (PERMANOVAs) on the effect of application of three doses of zinc (Zn) (0.1, 1, and 10 mg Zn plant^−1^) and plant compartment (shoot and root) on the expression of seven *MsZIP* genes and separately on the expression of other five genes (*MsZIF1*, *MsNAS1*, *MsHMA4*, *MsYSL1*, and *MsMTP1*). A PERMANOVA was also performed on the response of all the genes. The analysis of homogeneity of multivariate dispersion (PERMDISP) was also performed. The studied plant was alfalfa (*Medicago sativa* L.). The analysis also included no-Zn addition control.

Response Variables	Explanatory Variables	Zn Application (Zn)	Plant Compartment (Comp)	Zn x Comp	Residual
*ZIP* genes	Pseudo F	5.56	8.16	1.76	
	P(perm)	**0.002**	**0.001**	0.082	
	Explained variance (%)	29.1	22.9	9.7	38.3
	PERMDISP P(perm)	0.412	0.852		
Other genes	Pseudo F	3.06	5.59	1.76	
	P(perm)	**0.007**	**0.015**	0.1	
	Explained variance (%)	17.3	19.35	12.78	
	PERMDISP P(perm)	0.412	0.852		
All genes	Pseudo F	4.27	10.49	3.41	
	P(perm)	**0.001**	**0.001**	**0.003**	
	Explained variance (%)	17.3	25.2	25.6	31.9
	PERMDISP P(perm)	0.152	**0.030**		

**Table 2:** Gene name and forward and reverse sequences of 14 newly designed primer pairs for the quantification of the expression of genes of alfalfa (*Medicago sativa*) encoding proteins involved in cellular zinc (Zn) influx and efflux and Zn chelation. Two reference genes (i.e., *MsACT-101* and *MsEF1-α*) were also designed. The length of the amplicons, the primer amplification efficiency (%), and *R^2^* of the standard curve are indicated. The reference sequences are indicated by the accession number of the *Medicago truncatula* sequences and by the contig number of the *M. sativa* sequences. The primers were designed online Primer-Basic Local Alignment Search Tool (BLAST). See Figure 1 for the full names of the genes. Reference sequence accession number from NCBI—GenBank Accession number—https://www.ncbi.nlm.nih.gov/genbank/ (accessed on 2 March 2021). Reference sequence contig number from AGED—The Alfalfa Gene Index and Expression Atlas Database—http://plantgrn.noble.org/AGED/ (accessed on 2 March 2021).

Gene *	Reference Sequence Accession Number and Contig Number	Forward Primer (5′-3′)	Reverse Primer (5′-3′)	Amplicon Size (bp)	Efficiency (%)	*R^2^*
*MsZIP1*	AY339054 ^†^/19855 ^‡^	ATGATTAAAGCCTTCGCGGC	TCTGCTGGAACTTGTTTAGAAGG	233	99.8	0.999
*MsZIP2*	AY007281/82450	AGCCCAATTGGCGTAGGAAT	ACAGCAACACCAAAAAGCACA	215	99.3	0.999
*MsZIP3*	AY339055/33860	TGGTGTGATTTTGGCAACCG	TGACGGACCCGAAGAAACAG	325	104.9	0.999
*MsZIP4*	XM_003603101/92651	GGAGGGTGCATTTCTCAAGC	AGCAATGCCTGTTCCAATGC	108	97.1	0.999
*MsZIP5*	XM_013605712/66451	TGAAGGCATGGGACTTGGAA	CCAGCTGAAGCTGCATTGAA	192	99.3	0.998
*MsZIP6*	AY339058/9668	CTTGGCGACACGTTCAATCC	CCACAAGTCCCGAAAAGGGA	188	106.0	0.998
*MsZIP7*	AY339059/62098	GGCTTGTGCTGGTTATTTGAT	TTTCCATGCGTCTGCTTTTGT	310	96.1	0.999
*MsZIF1*	XM_003601836/59165	TGCCTGCATTTGGTTACCG	CTGCAGCTTCCACATTGTCAG	77	105.9	0.999
*MsHMA4*	XM_003626900/19210	TGCTCAACTTGCCAAAGCAC	GGAATGAACCATCCCAGCCA	111	108.9	0.999
*MsYSL1*	XM_024781439/4892	CAAGAAGCAAGTGCATGGGT	TCCACAGTCTTCTTTGCCTGAG	94	111.0	0.999
*MsMTP1*	FJ389717/67347	TGCAGCATTTGCCATCTCCT	TGCATAGAAACCAAAGCACCA	114	104.5	0.999
*MsNAS1*	XM_003594705/61146	GCTAGCTTGGCTGAAGATTGG	AGATACAAAGCACTCGGAGACA	87	100.5	0.999
*MsACT-101*	XM_003593074/89028	TCTCTGTATGCCAGTGGACG	TCTGTTAAATCACGCCCAGCA	140	102.4	0.999
*MsEF1-α*	XM_003618727/56897	CCACAGACAAGCCCCTCAG	TCACAACCATACCGGGCTTC	114	100.2	0.999

* See Figure 1 for the full names of the genes; ^†^ NCBI—GenBank Accession number—https://www.ncbi.nlm.nih.gov/genbank/ (accessed on 2 March 2021); ^‡^ AGED—The Alfalfa Gene Index and Expression Atlas Database—http://plantgrn.noble.org/AGED/ (accessed on 2 March 2021).

## Data Availability

The gene sequences and their annotations have been deposited in the NCBI under the submission # 2338923. The sequences of the obtained PCR amplicons have been deposited in the NCBI under the submission # 2338930.

## Supplementary Materials

The following are available online at https://www.mdpi.com/2223-7747/10/3/476/s1: Figure S1: Neighbor-joining (NJ) phylogenetic tree of ZIP gene sequences of alfalfa and other plant species. Figure S2: NJ phylogenetic trees of ZIF and MTP genes sequences ((a) and (b), respectively) of alfalfa and other plant species. Figure S3: NJ phylogenetic trees of YSL and HMA gene sequences ((a) and (b), respectively) of alfalfa and other plant species. Figure S4: NJ phylogenetic trees of NAS gene sequences of alfalfa and other plant species. Figure S5: Melting curve analysis of PCR products. Figure S6: Examples of electropherograms of PCR products of ZIP genes (*MsZIP1-7*). Figure S7: Examples of electropherograms of PCR products of the genes *MsZIF1*, *MsHMA4*, *MsYSL1*, *MsNAS1*, and *MsMTP1* and of the reference genes *MsACT-101* and *MsEF1-α*. Figure S8: Standard curves for the 14 newly designed primer pairs. Figure S9: Cycle threshold value of the reference genes. Table S1: Fresh and dry weight of shoots and roots of alfalfa after leaf Zn application. Material and methods S1: PCR details.
