# Supplementary material for: Transcriptional Regulation of Genes Involved in Zinc Uptake, Sequestration and Redistribution Following Foliar Zinc Application to Medicago sativa
Source: Plants (Basel). 2021 Mar 3;10(3):476. doi: 10.3390/plants10030476 (PMC7998959; doi:10.3390/plants10030476)
Supplement: Supplementary file 1 [file plants-10-00476-s001.pdf]

plants

MDPI

Supplementary Material

# Transcriptional Regulation of Genes Involved in Zinc Uptake, Sequestration and Redistribution Following Foliar Zinc Application to *Medicago sativa*

**Alessio Cardini [1,†], Elisa Pellegrino [1,†,*], Philip J. White [2], Barbara Mazzolai [3], Marco C. Mascherpa [4], and Laura Ercoli [1]**

[1] Institute of Life Sciences, Scuola Superiore Sant'Anna, Pisa, Italy; alessio.cardini@santannapisa.it (A.C.); elisa.pellegrino@santannapisa.it (E.P.); laura.ercoli@santannapisa.it (L.E.)
[2] Department of Ecological Science, The James Hutton Institute, Invergowrie, Dundee, United Kingdom
[3] Center for Micro-BioRobotics, Istituto Italiano di Tecnologia, Pontedera, Pisa, Italy; barbara.mazzolai@iit.it
[4] Istituto di Chimica dei Composti Organo Metallici, National Research Council (CNR), Pisa, Italy; marcocarlo.mascherpa@pi.iccom.cnr.it
† These authors contributed equally to this work
* Correspondence: elisa.pellegrino@santannapisa.it (E.P.)

# Supplementary Table

**Table S1.** Fresh and dry weight of shoots and roots of alfalfa (*Medicago sativa*) 5 days after the application of Zn doses of 0, 0.01, 0.1, 0.5, 1, or 10 mg Zn $plant^{-1}$ to leaves. Means ± standard error of three replicates are shown.

| Dose of Zn Application (mg $plant^{-1}$) | Shoot Fresh Weight | Root Fresh Weight | Shoot Dry Weight | Root Dry Weight |
|---|---|---|---|---|
| | g $plant^{-1}$ | | | |
| 0 | 4.28 ± 0.12 | 1.96 ± 0.46 | 0.75 ± 0.01 | 0.19 ± 0.01 |
| 0.01 | 3.85 ± 0.46 | 2.40 ± 0.06 | 0.63 ± 0.06 | 0.19 ± 0.01 |
| 0.1 | 4.73 ± 0.80 | 2.24 ± 0.16 | 0.84 ± 0.11 | 0.29 ± 0.04 |
| 0.5 | 4.27 ± 0.98 | 2.39 ± 0.24 | 0.78 ± 0.17 | 0.26 ± 0.03 |
| 1 | 4.07 ± 0.33 | 1.75 ± 0.19 | 0.78 ± 0.06 | 0.26 ± 0.01 |
| 10 | 4.29 ± 0.85 | 1.76 ± 0.10 | 0.82 ± 0.14 | 0.23 ± 0.05 |

## Supplementary Figures

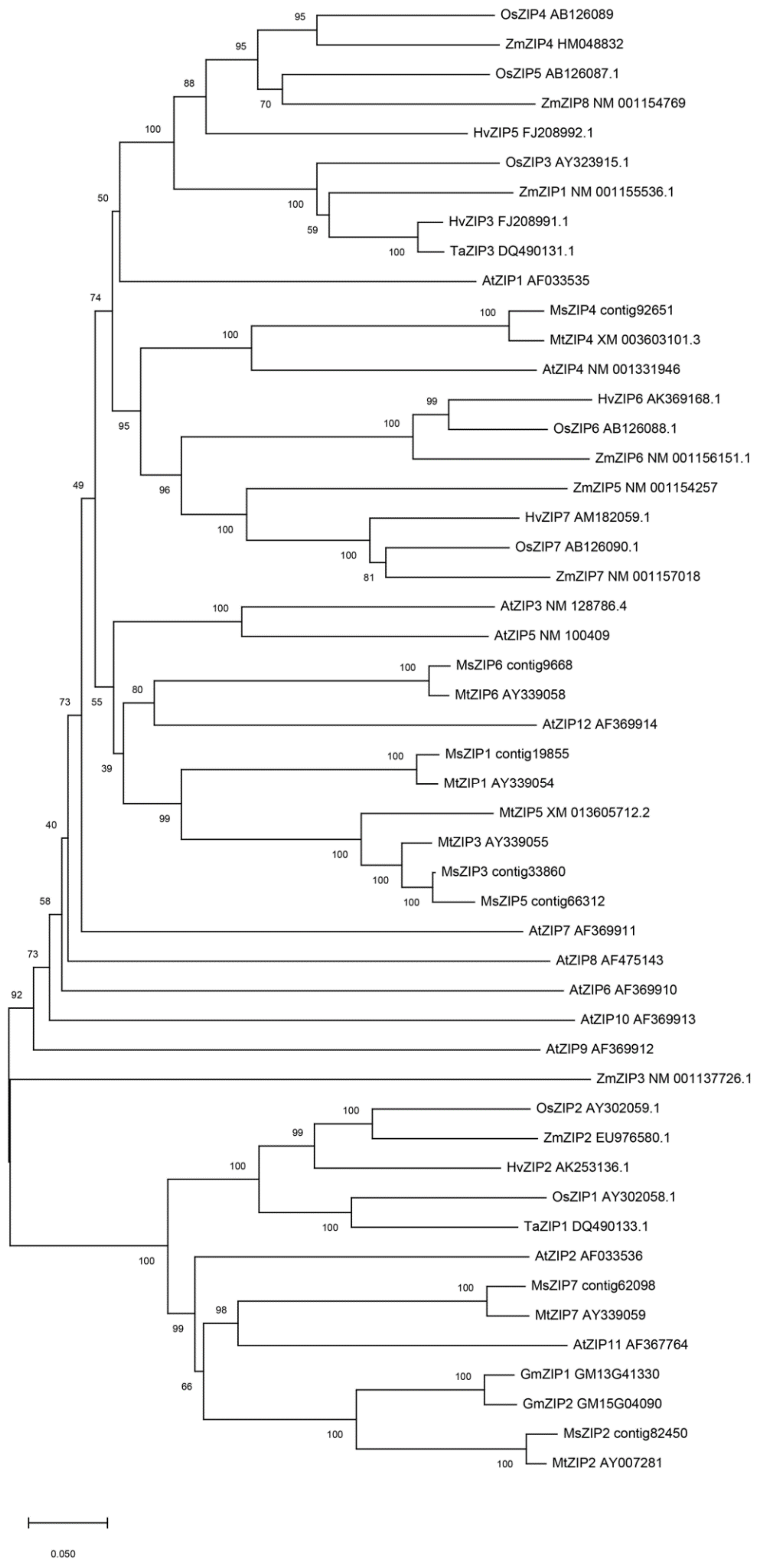

**Figure S1.** Neighbor-joining (NJ) phylogenetic tree (Saitou et al., 1987) of ZIP gene sequences of *Medicago sativa* L. and *Medicago truncatula* Gaertn. and of other plant species *(Arabidopsis thaliana* (L.) Heynh., *Glycine max* (L.) Merr., *Hordeum vulgare* L., *Oryza sativa* L., *Triticum aestivum* L., and *Zea mays* L.) obtained by GenBank search. The tree was inferred by MEGA X (Kumar et al. 2018), and evolutionary distances were calculated by the p-distance method (Nei and Kumar, 2000). Branch support bootstrap values were derived from 500 bootstrap replicates. The phylogram was drawn by MEGA X and edited by Adobe Illustrator CC 2017.

(a)

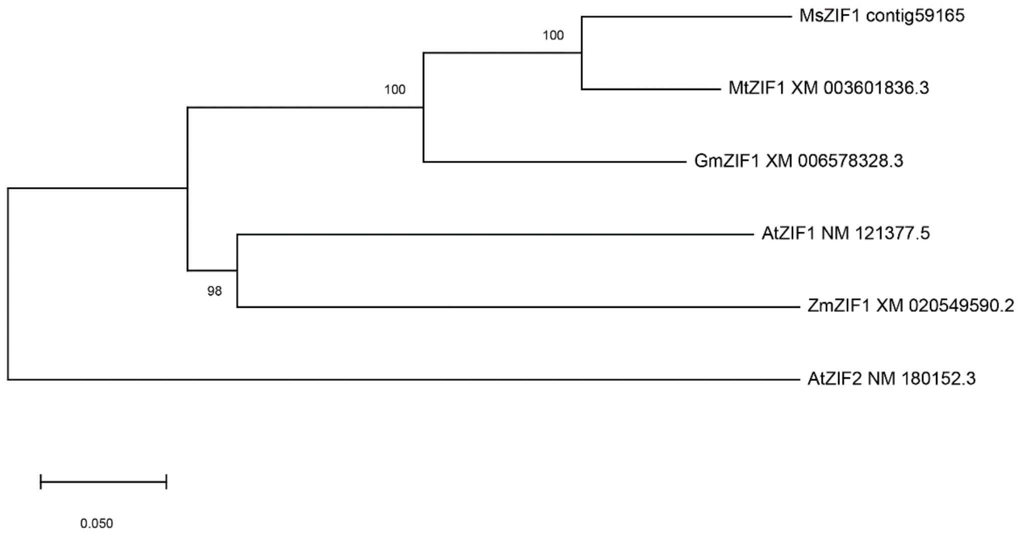


(b)

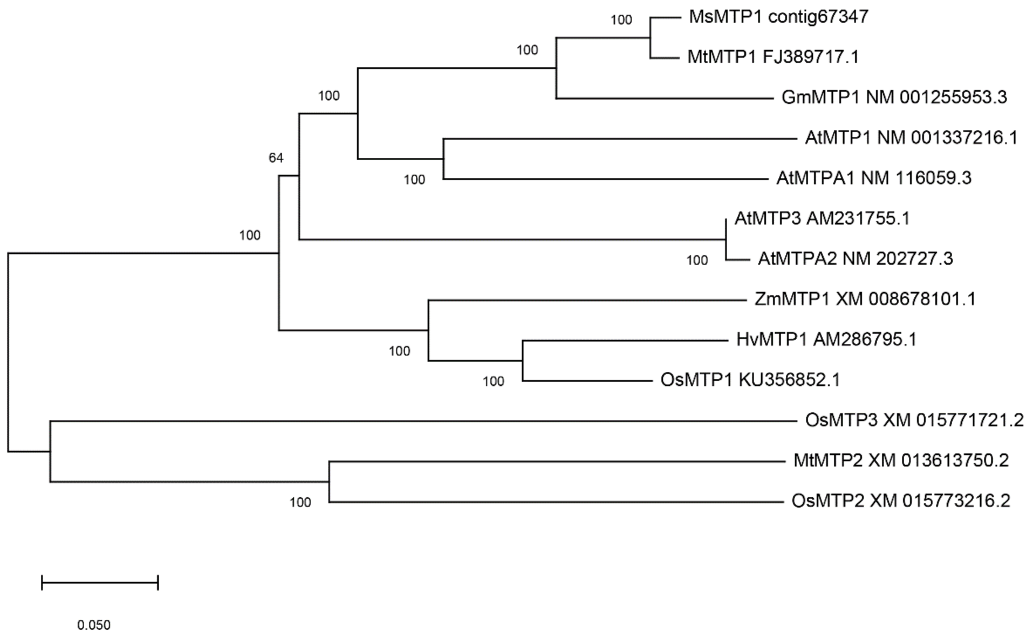


**Figure S2.** Neighbor-joining (NJ) phylogenetic trees (Saitou et al., 1987) of zinc-induced facilitator (ZIF) (a) and metal tolerance protein (MTP) (b); evolutionary distances were calculated by the p-distance method (Nei and Kumar, 2000). Branch support bootstrap values were derived from 500 bootstrap replicates. The phylograms were drawn by MEGA X and edited by Adobe Illustrator CC 2017.

(a)

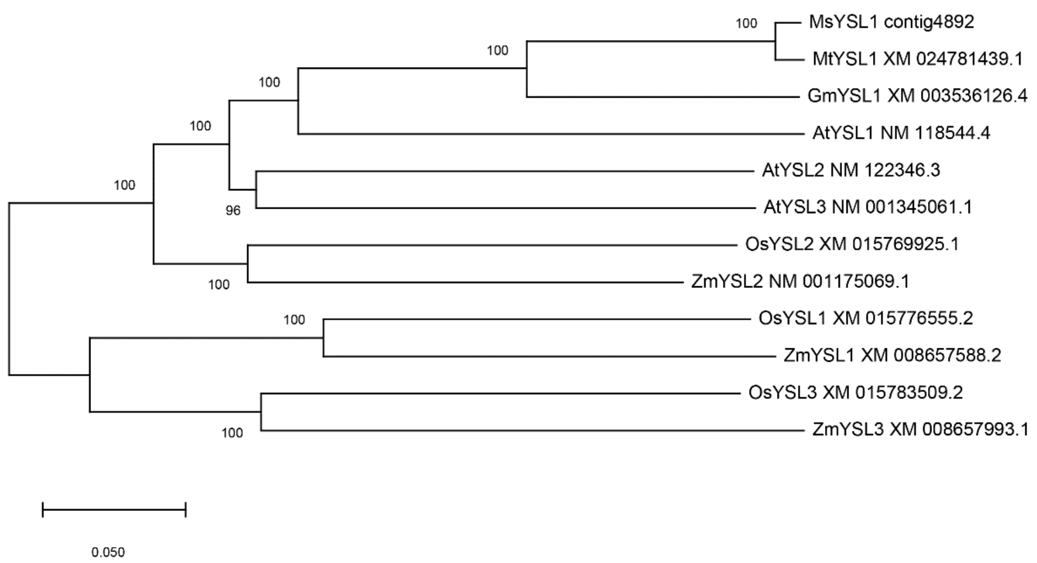
MsYSL1 contig4892
MtYSL1 XM 024781439.1
GmYSL1 XM 003536126.4
AtYSL1 NM 118544.4
AtYSL2 NM 122346.3
AtYSL3 NM 001345061.1
OsYSL2 XM 015769925.1
ZmYSL2 NM 001175069.1
OsYSL1 XM 015776555.2
ZmYSL1 XM 008657588.2
OsYSL3 XM 015783509.2
ZmYSL3 XM 008657993.1
0.050

(b)

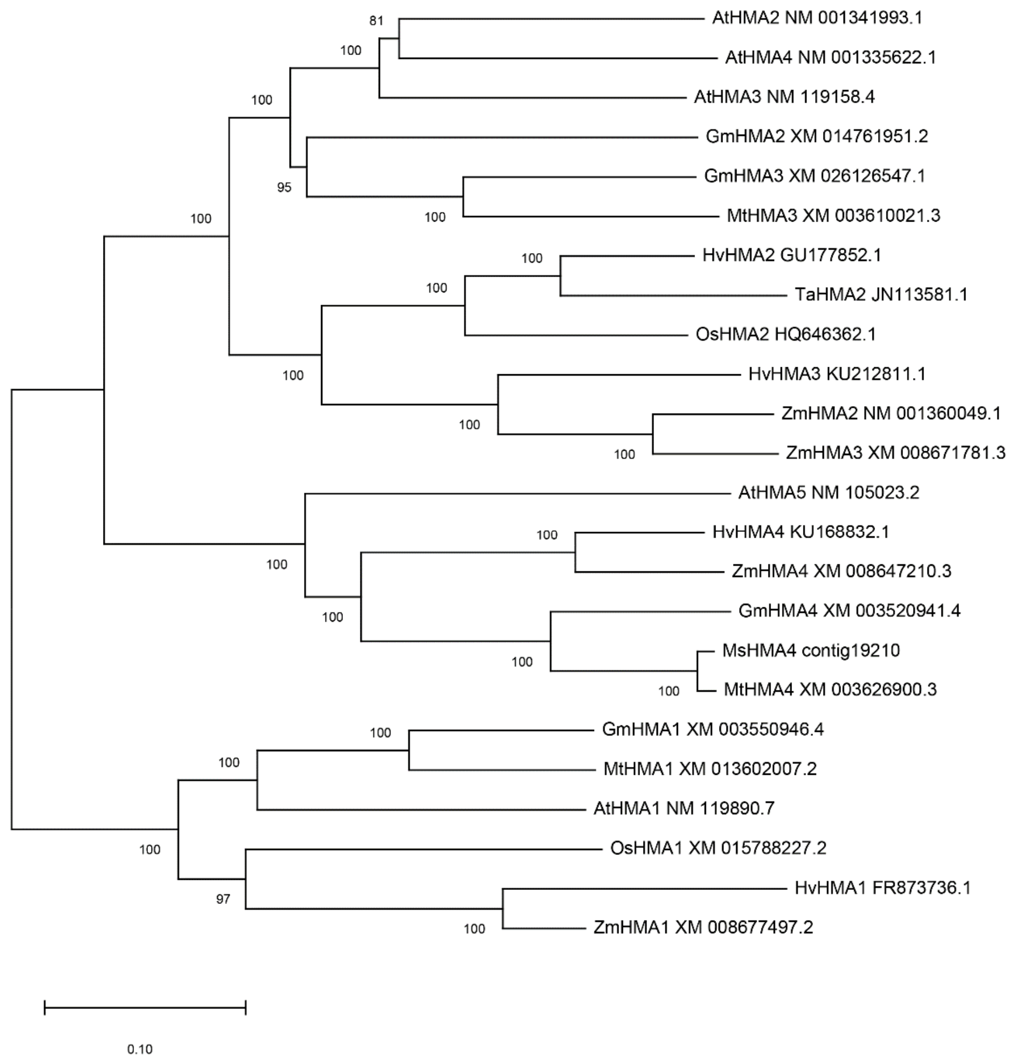
AtHMA2 NM 001341993.1
AtHMA4 NM 001335622.1
AtHMA3 NM 119158.4
GmHMA2 XM 014761951.2
GmHMA3 XM 026126547.1
MtHMA3 XM 003610021.3
HvHMA2 GU177852.1
TaHMA2 JN113581.1
OsHMA2 HQ646362.1
HvHMA3 KU212811.1
ZmHMA2 NM 001360049.1
ZmHMA3 XM 008671781.3
AtHMA5 NM 105023.2
HvHMA4 KU168832.1
ZmHMA4 XM 008647210.3
GmHMA4 XM 003520941.4
MsHMA4 contig19210
MtHMA4 XM 003626900.3
GmHMA1 XM 003550946.4
MtHMA1 XM 013602007.2
AtHMA1 NM 119890.7
OsHMA1 XM 015788227.2
HvHMA1 FR873736.1
ZmHMA1 XM 008677497.2
0.10

**Figure S3.** Neighbor-joining (NJ) phylogenetic trees (Saitou et al., 1987) of yellow stripe-like protein (YSL) (a) and heavy metal transporter (HMA) (b) gene sequences of *Medicago sativa* L. and *Medicago truncatula* Gaertn. and other plant species (*Arabidopsis thaliana* (L.) Heynh., *Glycine max* (L.) Merr., *Hordeum vulgare* L., *Oryza sativa* L., *Triticum aestivum* L., and *Zea mays* L.) obtained by GenBank search. The trees were inferred by MEGA X (Kumar et al. 2018), and evolutionary distances were calculated by the p-distance method (Nei and Kumar 2000). Branch support bootstrap values were derived from 500 bootstrap replicates. The phylograms were drawn by MEGA X and edited by Adobe Illustrator CC 2017.

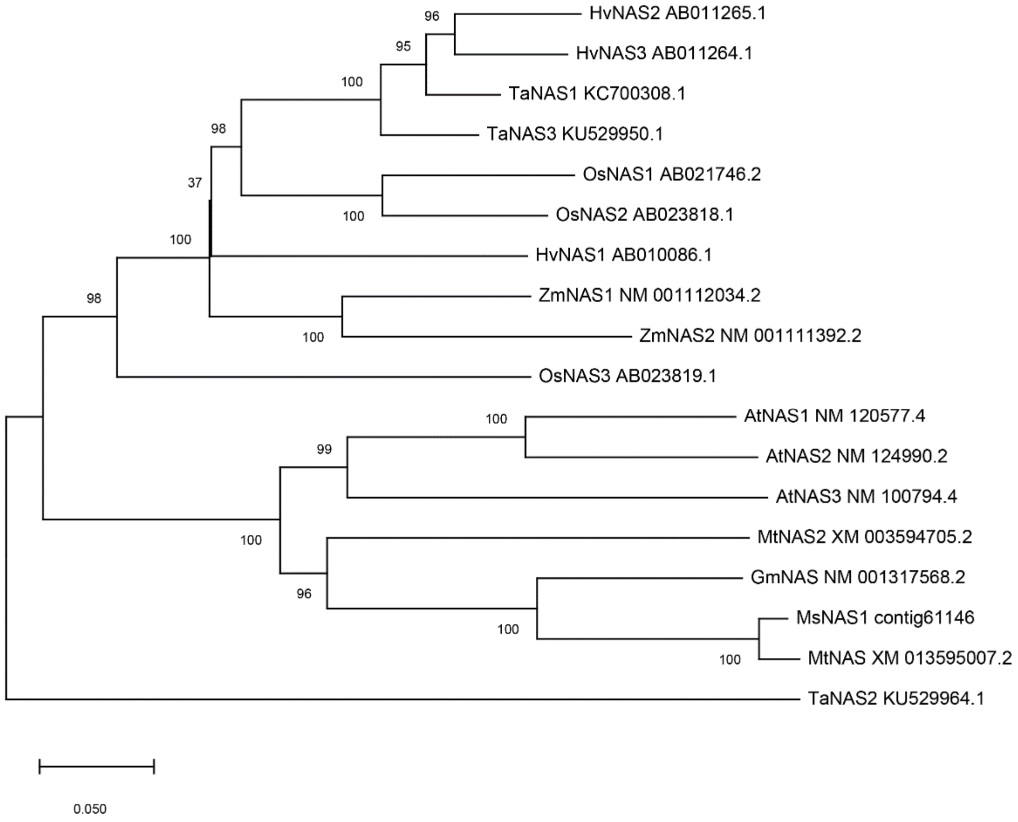


**Figure S4.** Neighbor-joining (NJ) phylogenetic trees (Saitou et al., 1987) of nicotianamine synthase (NAS) gene sequences of *Medicago sativa* L. and *Medicago truncatula* Gaertn. and other plant species (*Arabidopsis thaliana* (L.) Heynh., *Glycine max* (L.) Merr., *Hordeum vulgare* L., *Oryza sativa* L., *Triticum aestivum* L., and *Zea mays* L.) obtained by GenBank search. The trees were inferred by MEGA X (Kumar et al. 2018), and evolutionary distances were calculated by the p-distance method (Nei and Kumar, 2000). Branch support bootstrap values were derived from 500 bootstrap replicates. The phylograms were drawn by MEGA X and edited by Adobe Illustrator CC 2017.

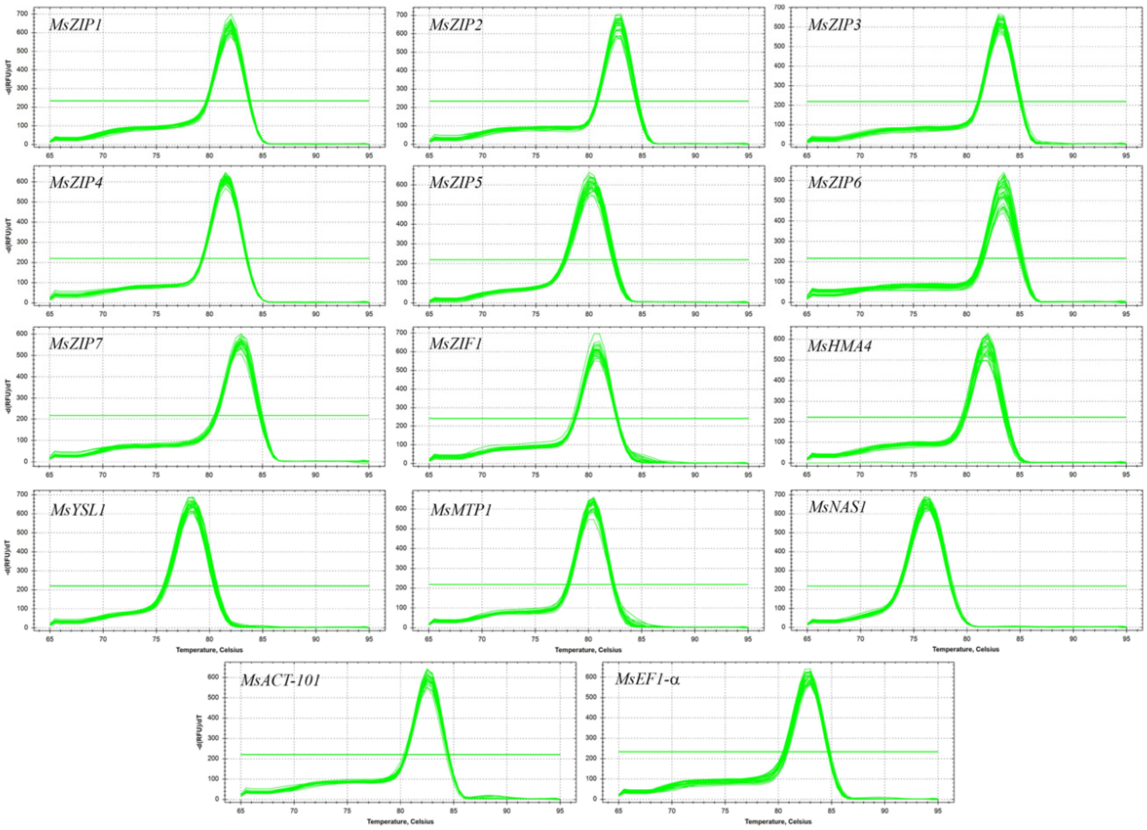


**Figure S5.** Melting curve analysis of the PCR products obtained by the newly designed 14 pair of primers: *MsZIP1–7* transporters and other Zn-related genes (i.e., *MsZIF1*, *MsHMA4*, *MsYSL1*, *MsNAS1*, and *MsMTP1*).

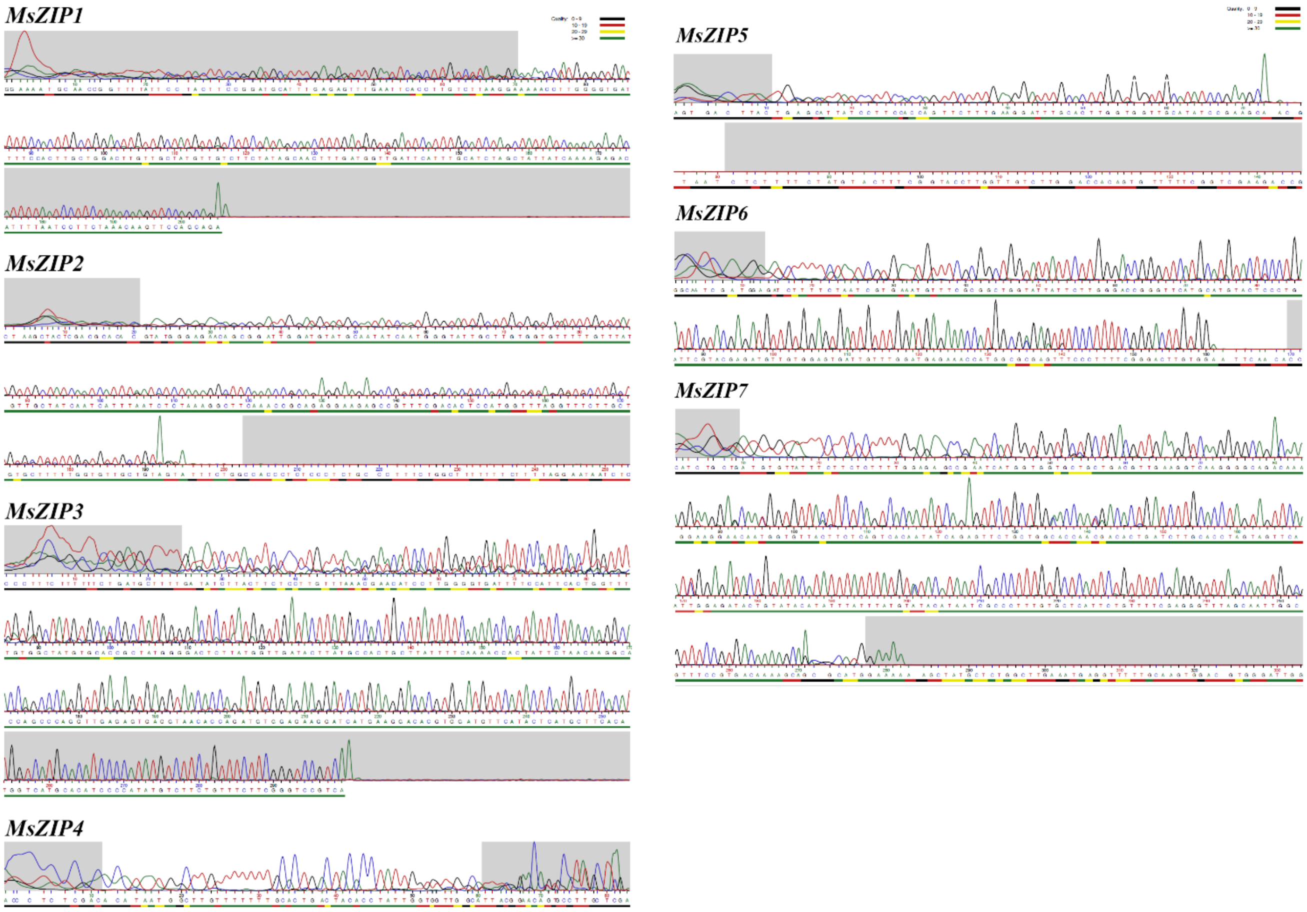


**Figure S6.** Examples of electropherograms of seven PCR products obtained by the newly designed primers for MsZIP1–7 transporters. The electropherograms represent one replicate of the three PCR products obtained by each primer pair.

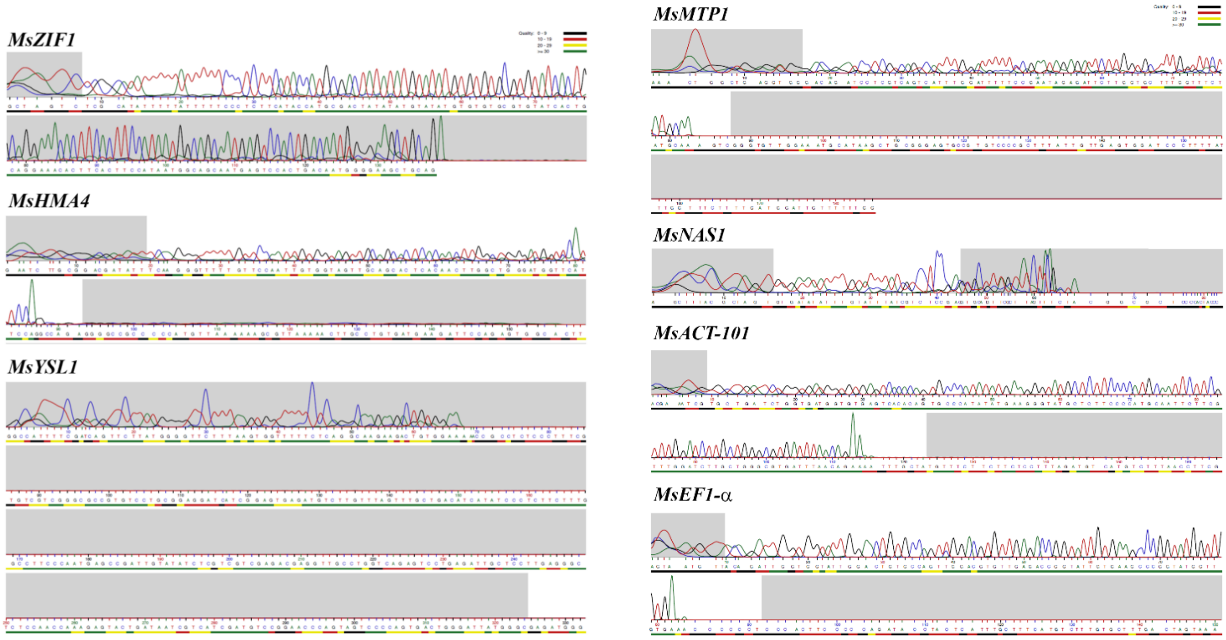


**Figure S7.** Examples of electropherograms of seven PCR products obtained by the newly designed primers for genes involved in Zn transport-related processes: *MsZIF1*, *MsHMA4*, *MsYSL1*, *MsNAS1*, and *MsMTP1*. The electropherograms of the PCR products obtained by the newly designed reference genes, *MsACT-101* and *MsEF1-α*, are also reported. The electropherograms represent one replicate of the three PCR products obtained by each primer pair.

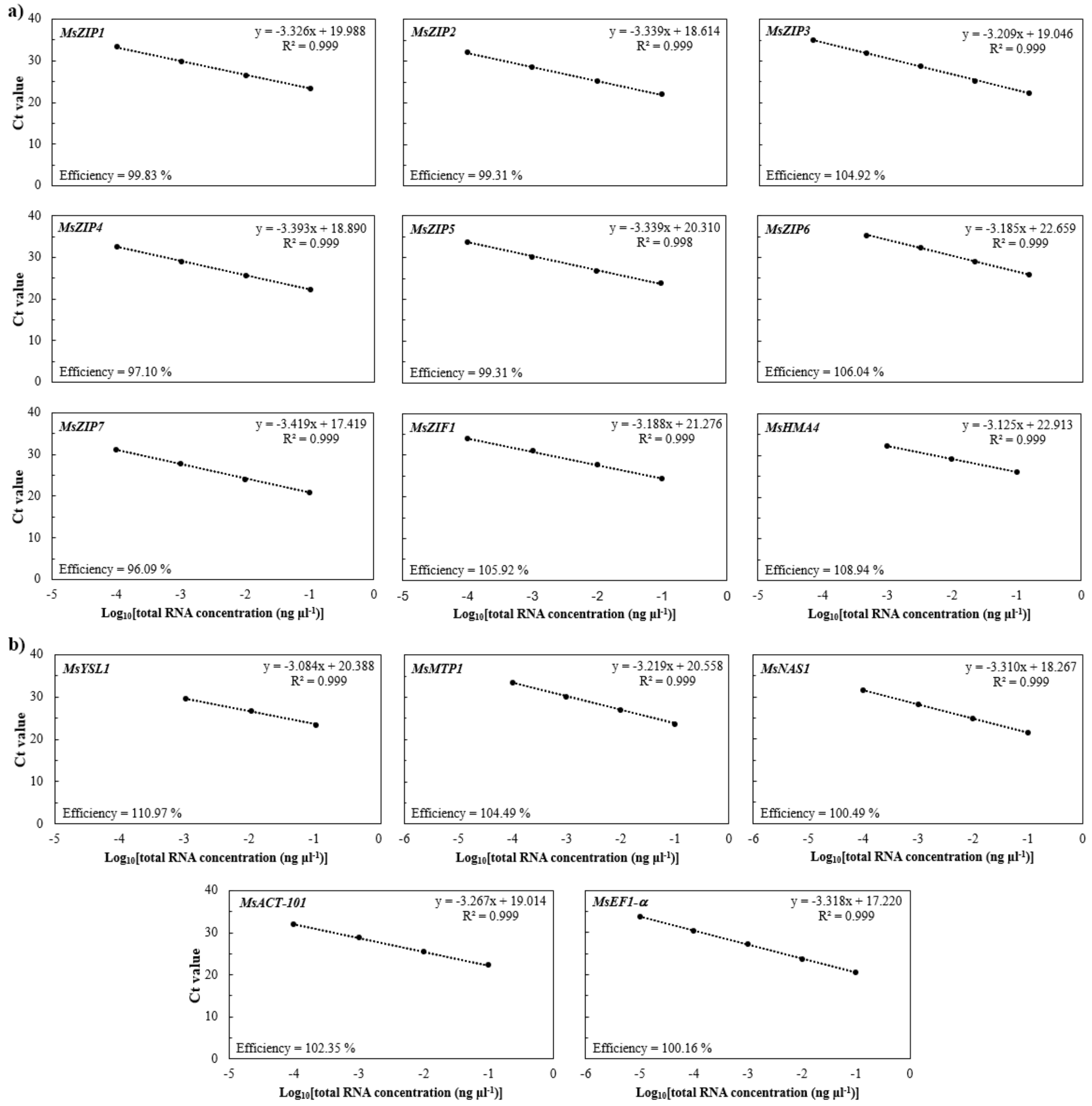


**Figure S8.** Standard curves for 14 newly designed primer pairs (a,b). The curves are based on three replicates of 10-fold serial dilutions of complementary DNA (cDNA). The cycle threshold values (Ct) are plotted against the decimal logarithms of the concentrations of the reverse-transcribed total RNA. Linear regressions are shown in each subfigure in order to indicate the linear range of quantification and the precision ($R^2$). The amplification efficiency (efficiency), as inferred from the slope of the lines (S: angular coefficient), is indicated at the bottom left of each subfigure (%). The names of the genes (abbreviations) and localization are shown in Figure 1.

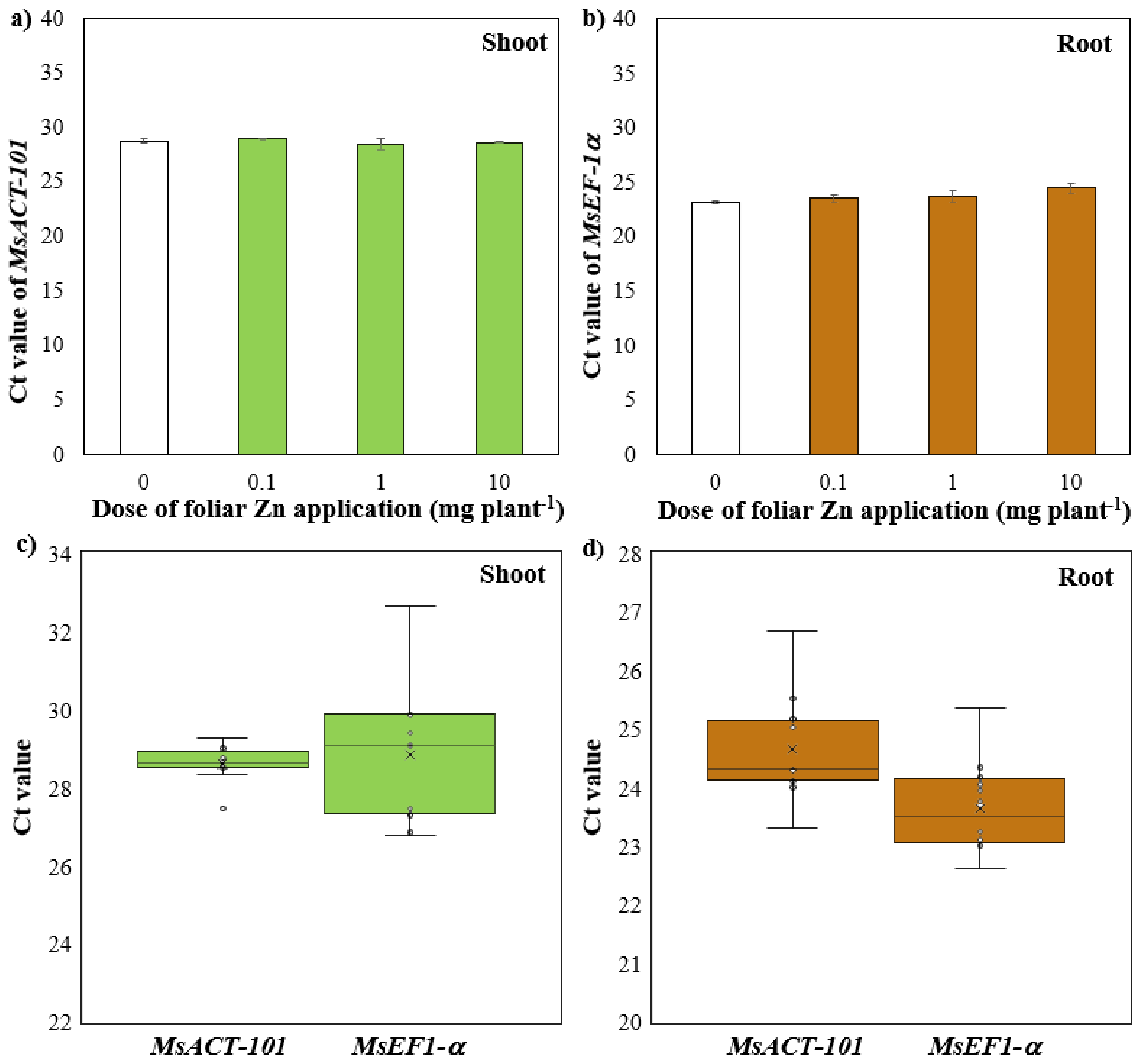


**Figure S9.** Cycle threshold (Ct) value of the reference genes, actin 101 (*MsACT-101*), and elongation factor 1-*α* (*MsEFf1-α*) in shoots (**a**,**c**) and roots (**b**,**d**) for the no-Zn addition control and the three Zn doses (0.1, 1, 10 mg Zn plant$^{-1}$). The Ct values of *MsACT-101* in shoots and of *MsACT-101* in roots were stable across the Zn doses, as confirmed by the non-significant ANOVA (data not shown).

## References


1. Kumar, S.; Stecher, G.; Li, M.; Knyaz, C.; Tamura, K. MEGA X: Molecular evolutionary genetics analysis across computing platforms. *Mol. Biol. Evol.* **2018**, *35*, 1547–1549, doi:10.1093/molbev/msy096.
2. Nei, M.; Kumar, S. Molecular evolution and phylogenetics. Oxford University Press: Oxford, United Kingdom, 2000.
3. Saitou, N.; Nei, M. The neighbor-joining method: A new method for reconstructing phylogenetic trees. *Mol. Biol. Evol.* **1987**, *4*, 406–425, doi:10.1093/oxfordjournals.molbev.a040454.

**Supplementary Material and Methods.**

**Material and Methods S1.**

**PCR Details.**

PCR amplifications were performed using the designed primer pairs (3 replicates per each PCR amplification), targeting the selected genes. PCR amplicons were generated in volumes of 20 μL with 0.5 U of HotStarTaq DNA Polymerase (Qiagen, Venlo, Netherlands), 10 μM of each primer, 0.2 mM of each dNTP, 1 mM of $MgCl_2$, and 1x reaction buffer, using a S1000 Thermal Cycler TM (BIORAD, Hercules, CA, USA). The thermal cycler was programmed as follows: 95 °C for 2 min, 30 cycles at 95 °C for 45 s, 60 °C for 45 s, 70 °C for 1 min, and a final extension step at 72 °C for 10 min. Reaction yields and fragment lengths were estimated using a 1% agarose gel electrophoresis, containing Sybr Safe (Invitrogen, Carlsbad, CA).
